# Supplementary material for: Complexome profiling of the Chlamydomonas psb28 mutant reveals TEF5 as an early PSII assembly factor
Source: Plant Cell. 2025 Mar 17;37(6):koaf055. doi: 10.1093/plcell/koaf055 (PMC12164590; doi:10.1093/plcell/koaf055)
Supplement: koaf055_Supplementary_Data [file koaf055_supplementary_data.zip › psb28_interactive.zip!/plots/intensity/qualityPlots/peptides/DLE2_Cre11.g468450.t1.2.html]

### Cre11.g468450.t1.2

Trivial name: DLE2  
SeqCoverage [median]: 31.40  
Mass: 19.46 kDa  
  
 

| Sequence | MissedCleavage | Mass [Da] | wtIntensity | muIntensity | Score |
| --- | --- | --- | --- | --- | --- |
| ALGFEPK | 0 | 760.41 | 2160.53 | 0.00 | 76.85 |
| EAFDLFDTDGSGTIDAK | 0 | 1800.81 | 905.64 | 0.00 | 89.63 |
| LFDDDNSGTITIK | 0 | 1437.70 | 2471.25 | 6045.93 | 116.77 |
| LFDDDNSGTITIKDLRR | 2 | 1978.01 | 584.54 | 1516.88 | 62.38 |
| VGLTEEQKQEIR | 1 | 1428.76 | 5144.06 | 1533.65 | 70.41 |
|  |  |  | 11266.0 | 9096.4 |  |
